# Impact of COVID-19 on Periodontitis and Peri-Implantitis: A Narrative Review

**DOI:** 10.3389/froh.2022.822824

**Published:** 2022-02-10

**Authors:** Leonardo Mancini, Lorenzo Maria Americo, Tommaso Pizzolante, Raffaele Donati, Enrico Marchetti

**Affiliations:** ^1^Department of Life, Health and Environmental Sciences, University of L'Aquila, L'Aquila, Italy; ^2^PerioAQ Group, Clinica Odontoiatrica Delta Sei, L'Aquila, Italy; ^3^Department of Experimental and Clinical Medicine, School of Dentistry, University of Florence, Florence, Italy

**Keywords:** COVID-19, periodontitis, peri-implantitis, cytokines, inflammation, ACE-2, bone remodeling

## Abstract

COVID-19 is reported as one of the most widespread diseases in the world. An extraordinary number of articles and manuscripts have focused on the inflammatory cascade and sequelae, showing the important roles of cytokines and renin-angiotensin levels and possible links to other pathologies. Nowadays, interest regarding the possible correlation between COVID-19 and periodontal and Peri-implant diseases is increasing. This mini-review aims to collect data regarding the possible correlation between COVID-19 and periodontitis or Peri-implantitis through the analysis of articles published in the last 3 years. The following keywords were used: ([periodontitis OR periodontal disease] AND [COVID-19]); ([Peri-implantitis OR mucositis] AND [COVID-19 OR Sars-CoV-2]). The inclusion criteria were studies on COVID-19 or SARS-CoV-2 and periodontitis or Peri-implantitis, and studies on the molecular and cellular aspect of COVID-19 in periodontal or Peri-implant tissues. The search revealed 484 articles in total (PubMed 208 and Scopus 276). After a screening of titles and abstracts, 47 articles were included in the full-text analysis. Two articles comprised the Peri-implant group: a short communication and a review. Regarding the periodontal group, 45 articles were selected and analyzed according to the type of study, population, and aim. Of these, 10 articles were clinical studies, and the other 35 were hypotheses, reviews, letters to the editor, or commentaries. In conclusion, according to the data extracted, a mutual correlation between COVID-19 and periodontitis can be stated; however, data linked to Peri-implantitis are still missing, and future clinical studies are still needed.

## Introduction

Periodontal and Peri-implant diseases and Coronavirus 2019 (COVID-19) are three pathologies with different onsets and origins [1–3]. Periodontitis and Peri-implantitis are inflammatory conditions linked to the presence of bacteria, biofilms, and other factors related to susceptibility and genetic inheritance [3–6]. On the other hand, COVID-19 is a viral disease related to a strong cytokine storm syndrome where patients are overwhelmed by multi-organ disease. The oral cavity and lungs are involved with the presence of heavy pneumonia, which can lead to death in many cases [7–10]. The effect of this pathology in the oral cavity has aroused increasing interest during the last 2 years. Several hypotheses, reviews, and case-series have been published attempting to correlate these three diseases where the inflammatory pattern is common [11–14]. According to a recent scoping review, no conclusion was possible due to missing clinical studies on this topic [15]. Nevertheless, the data collected focused on the role of angiotensin-converting enzyme-2 (ACE-2) in facilitating the entrance of SARS-CoV-2 into the organism and at the same time inducing activation of an inflammatory cascade as mentioned also in a hypothesis published by Mancini et al. in 2020 [15, 16]. This inflammatory pattern, as reported in a clinical study, might be accentuated in the presence of periodontitis [17]. According to Marouf et al. [17], periodontal patients have a high risk of complications from COVID-19. Furthermore, as reported in a case-control study by Anand et al. [18], COVID-19 patients are more susceptible to gingivitis, plaque accumulation, and bleeding. The link between Peri-implantitis and COVID-19 was a hypothesis already published 2 years ago according to Kadkhodazadeh et al. [19], where the inability to attend follow-up visits might make patients more susceptible to plaque accumulation and bleeding. Nevertheless, a real and concrete clinical correlation was not revealed. This review aimed to collect data and summarize possible correlations, hypotheses, and clinical reports regarding the links between periodontitis, Peri-implantitis, and COVID-19.

## Materials and Methods

A literature search was performed in two large electronic databases (PubMed and Scopus) with the following keywords ([periodontitis OR periodontal disease] AND [COVID-19]); ([Peri-implantitis OR mucositis] AND [COVID-19 OR Sars-CoV-2]). The last search was conducted on 31 October 2021.

*The inclusion criteria were the following:*

- Studies which tried to link COVID-19 and periodontitis or Peri-implantitis
- Studies on the molecular and cellular aspects of COVID-19 in periodontal or Peri-implant tissues.

Any type of study design was included in order to collect as much data as possible regarding this possible correlation.

*The exclusion criteria were:*

- Studies published in languages other than English
- Studies that did not assume a correlation.

### Selection Process

The selection of studies for inclusion was conducted by two reviewers (LM and TP). After the removal of all duplicates, the remaining articles were selected and included according to a title and abstract check. In case of disagreement, a third reviewer (EM) was invited to check the article. In the end, a full-text analysis of the remaining manuscripts was performed. The agreement between the reviewers was scored according to Cohen's kappa evaluation.

### Data Extraction

The data from the eligible articles were extracted and handled by three reviewers (TP, LAM, and RD). An Excel spreadsheet (Microsoft Corporation; Redmond, USA) was generated in order to collect data regarding the type and time of publications, the aim of the studies, population, mechanism of correlation, and possible bias reported in the articles. Before starting the extraction, a calibration between the reviewers was performed, reducing possible missing data. Thus, three articles were used for calibration and a fourth reviewer expert in the field of systematic reviews (LM) was in charge of checking the tables from each reviewer.

## Results

**Figure 1:**
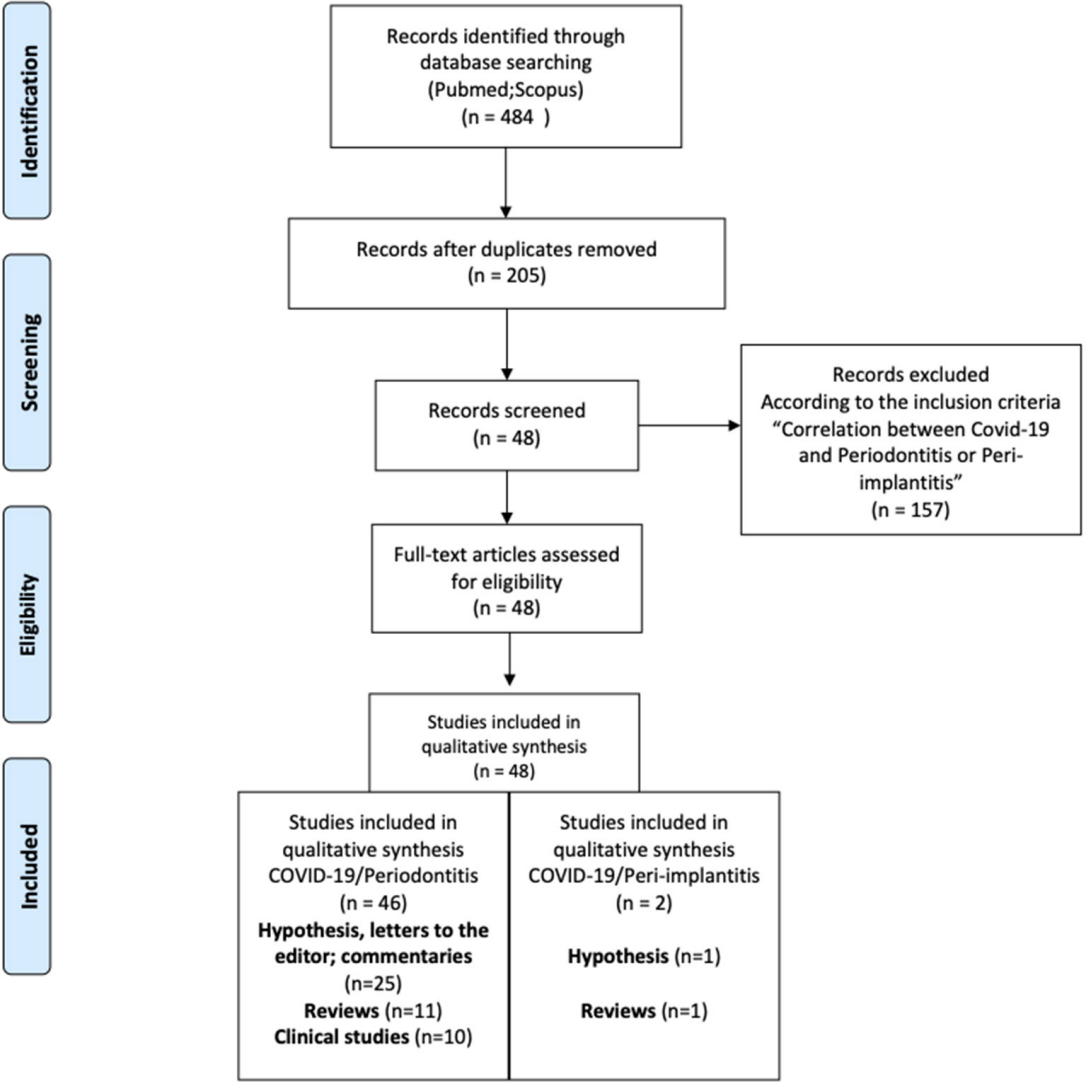
Flow chart for the inclusion and exclusion process.

“Forty-seven” articles were included for data extraction, and the agreement between the readers was favorable (*K* = 0.94). Forty-five articles linked COVID-19 to periodontitis, while only 2 described the possible correlation between COVID-19 and Peri-implantitis. Figure 1 shows the flow chart of the review with all the screening phases.

### Periodontitis and COVID-19

Most of the articles analyzed suggested the existence of a possible relationship between COVID-19 and periodontal disease. Three main mechanisms of action seemed to be more closely involved in this report.

- The presence of pathogenic bacteria in the oral cavity of periodontal patients may result in their aspiration and expose periodontal patients with COVID-19 to an increased risk of complications; [20–23].
- The inflammatory state that characterizes patients with periodontal disease, associated with the inflammation generated by COVID-19, can more easily induce aggravation of the disease status of patients and leads to complications associated with COVID-19 infection [15, 18, 23–30].
- Several articles refer to the possibility that patients with periodontal disease are more susceptible to COVID-19 infection because they already have over-expression of the ACE-2 receptor, implicated in the entry of SARS-CoV-2 in the host [16, 21, 23, 28, 31–41].

#### Hypotheses, Letters to the Editor, and Commentaries

“Twenty-four” articles were included showing the possible interactions between periodontitis and COVID-19, additional data are available in Supplementary Table 1:

- Receptors have an important role in SARS-CoV-2 entry and infection, and ACE-2 was considered by many articles as the main receptor in the oral cavity and other tissues such as the pancreas and salivary glands [16, 21, 31–33, 38, 41–45]. The cluster of differentiation 147 (CD147) is considered as another main coronavirus receptor [31, 42]. The presence, especially in salivary glands, of Transmembrane protease, serine 2 (TMPRSS2) has been depicted as an important factor that facilitates SARS-COV-2 entry [25, 43].
- Poor oral health conditions and pathologies, like periodontitis and oral cancer, could contribute to the aggravation of COVID-19 infection [45]. In contrast, improving oral health could reduce the incidence of SARS-CoV-2, as well as COVID-19 aggravation and mortality [13, 33].
- A role for cytokine release in the correlation between COVID-19 and periodontitis has been hypothesized in several articles. The pro-inflammatory storm can promote adhesion and infection in the respiratory tract after the aspiration of periodontal pathogens [13, 21]. In addition, it has been observed that the inflammatory response, and thus cytokine release, may aggravate COVID-19 conditions. Moreover, the cytokine storm released by COVID-19 is similar to the one released by periodontitis [24, 25, 29–31, 37, 46]. Lastly, high levels of interleukin 6 (IL-6) and interleukin 17 (Il-17) have been associated with concurrent COVID-19 and periodontal disease [29, 31].
- In patients with periodontal disease, there is an increased protease level that may aggravate and enhance COVID-19 infection [43]. Mainly, in the different articles, the following types of proteases were analyzed, and it was observed that a high level of furin [42, 43, 45], cathepsin B [43], cathepsin L [43, 45], and cathepsin G may increase the risk of COVID-19 complications [45].
- Some articles have focused on the role of diabetes in the association between COVID-19 and periodontitis. It has been observed that COVID-19 may lead to diabetes, salivary alteration, and subsequently, periodontal disease [41], but diabetes can also lead to periodontitis, and it may increase the risk of pneumonia and COVID-19 [33]. All these common factors may also explain better the reason why periodontal disease and COVID-19 share many risk factors [47].
- Neutrophil extracellular traps (NET) are increased and play a key role in COVID-19 and periodontitis [30, 32]. It has also been noted that a higher level of CD14+ and/or CD16+, due to periodontitis, could enhance COVID-19 aggravation [45].
- Galectin-3 and melatonin were molecules that seem to be directly linked to periodontal disease severity and COVID-19 infection [43, 44, 48].
- Also, cannabis has been taken into account, it could have both a positive and a negative effect on the periodontium and thus on the risk of COVID-19 infection, too [49].
- COVID-19 may lead to a higher risk of periodontal conditions, such as necrotizing periodontal disease (NPD) [50].
- Active metalloproteinase-8 (aMMP-8) may be found in patients with COVID-19 and periodontitis [51].

#### Reviews

From the screened reviews (Supplementary Table 2), the following main findings were extracted:

Poor oral hygiene, and the inhalation of periodontal bacteria may aggravate COVID-19 infection, and thus the improvement of oral health may reduce COVID-19 complications [20, 22, 23, 27].

Appropriate oral hygiene and mouth rinse use are mandatory to prevent the spread of COVID-19 [19, 20].

Inflammation, and thus increases in cytokines (such as IL1, IL6, IL8) typical of periodontitis, is the link with COVID-19; it may also aggravate the conditions of COVID-19 infection [23, 26, 28, 52].

Periodontal disease could aggravate the cytokine storm syndrome in COVID-19 patients, and according to Basso and Sukumar, the cytokine storm could be supported by a massive production of periodontal biofilm [15, 27].

Furthermore, according to Basso et al., periodontal bacteria increase the expression of ACE-2 [15]. Another clinical aspect was suggested by Casillas Santana et al., that uncontrolled hyperglycemia in diabetic patients could increase the expression of ACE-2, reducing the activity of MMP [36].

Crevicular fluid and periodontal pockets may act as a niche for SARS-CoV-2 [15, 35, 39].

COVID-19 may produce oral pain and desquamative gingivitis, and damaged oral mucosa is an open door for the virus [35, 52].

Periodontal treatment might decrease the viral load in the periodontal pocket and also the expression of Furin and Cathepsin L (which are responsible for viral infection), whereby, in accordance with Bertolini et al., we could consider periodontal treatment for the clinical management of COVID-19 patients [39].

#### Clinical Studies

A mutual correlation was underlined among a few of the included studies; periodontal patients are more susceptible to COVID-19 and thus a strict and detailed periodontal treatment plan needs to be assessed [17, 18, 53, 54]. According to Marouf et al., COVID-19 complications in periodontal patients with stages 2–4 were reported with an odds ratio (OR) of 6.34 (2.79–14.61) OR for the death of periodontal patients (stage 2–4), affected by COVID-19, was 17.5 (2.27–134.8). Periodontal parameters were monitored and compared in another study according to Anand et al. [18] and in a case-control study patients after COVID-19 showed an increase in each periodontal parameter as probing depth (PD) of 2.09 ± 0.48 (Mean ± SD) compared to the control group 1.48 ± 0.36. Bleeding on probing (BoP) was higher in the case group compared to the control, respectively, 0.62 ± 0.24 and 0.29 ± 0.20. Interesting data were published by Gupta et al. [54], showing that males seem to be more susceptible to COVID-19 than females. Nevertheless, according to the data from Gupta et al. [54, 55], the assessment of a direct correlation was not possible.

Moreover, according to two articles, it seems that there is a lack of evidence supporting the claim that periodontitis is linked to a higher risk of COVID-19 infection [53, 54]. i Interesting data were published in a longitudinal cohort study where periodontitis, obesity and COVID-19 were examined and obesity seems to have a more impact on hospitalization than periodontitis for COVID-19 patients [56].

Periodontal disease seems to be associated with a higher risk of COVID-19 complications, including intensive care unit (ICU) admission, assisted ventilation, death, and serum increase in markers of COVID-19 worsening, including D-dimer, white blood cells (WBC), and C-reactive protein (CRP) [17].

Diabetes and periodontitis increase the expression of ACE-2 where ACE-2, TMPRSS2, and FURIN (highly expressed in the epithelial cells of the oral mucosa) play a crucial role in COVID-19 invasion [34, 40, 57]. A detailed explanation of the studies was reported in Table 1.

**Table 1:** Clinical studies included in the review, data regarding type of study, population outcome, and details for the possible correlations are listed.

**References**	**Type of article**	**Population**	**Outcomes**	**Limitations reported in the studies**	**Inclusion criteria**	**Exclusion criteria**	**SARS-CoV-2 entry mechanism**
Anand et al. [18]	Case-control study	COVID-19 positive group 79 control group 71	• PI ≥ 1:19 case/3 control • BoP: 74 case/ 36 control • CAL ≥ 2 mm: 51 case/5 control • Severe periodontitis: 39 case/7 control	Periodontal screening was recorded after a negative test for COVID-19.	Case group: patients with positive rRT-PCR control group: patients with negative rRT-PCR both groups: age ≥ 18, teeth ≥ 20	NR	Using the ACE-2 receptor.
Gupta et al. [54]	Cross-sectional analytical study	Total 82 (48 M/34 F)	• Association between necessity of oxygen in COVID-19 positive patients and BoP, increased periodontal probing depth, presence of gingival recession, and CAL. • Link between compromised patients with periodontal disease and death. • Link between patients with periodontal disease, COVID-19, and hospitalization.	Can't find a causal relationship; small sample size.	NR	Pregnant women; age <18; unwilling or not in a position to give written informed consent.	NR
Gupta et al. [55]	Clinical study	Total 33 (19 M/14 F COVID-19 positive patients	• Statistically insignificant association among COVID-19 and periodontitis or oral clinical manifestations. • 17 patients show oral clinical manifestations. • COVID-19 identified in 64.52% of saliva samples and 63.64% of GCF samples. • SARS-CoV-2 levels in GCF and saliva were comparable.	• Small sample size. • Temporal associations not evaluable.	NR	NR	COVID-19 detected in GCF.
Larvin et al. [56]	Retrospective	Patients tested for COVID-19 (*n* = 13,502)	• Evidence was insufficient to assess that periodontal disease is linked to an increased risk of COVID-19 infection, but the risk of mortality was higher in patients that also had periodontal disease.	• Information about hospital admission and mortality were delayed. • Self-reported oral health status as indicators of periodontal disease. • No information about past periodontal treatment. • Short study follow-up time.	• Patient with self-reported periodontal disease: painful and bleeding gums as indicators of mild-moderate periodontal disease. • Lost teeth as an indicator of severe periodontal disease.	NR	NR
Larvin et al. [53]	Longitudinal cohort study	Patients tested for COVID-19 (*n* = 58,897)	• The impact of obesity on COVID-19 is worse than the impact of periodontitis.	• Reduced specificity and sensitivity in the self-reported oral health indicators. • No random sampling. • Low prevalence of periodontal disease in the study sample.	• Painful and bleeding gums as indicators of mild-moderate periodontal disease. • Lost teeth as an indicator of severe periodontal disease.	NR	NR
Marouf et al. [17]	Case-control study	Total 568 Periodontal patients 258; control patients 310 (discharged without major complications)	• Association between periodontitis and severity of COVID-19. • Possible association among periodontitis and need for ventilation. • WBC and CRP serum markers were significantly higher in patients with COVID-19 who also had periodontitis.	• Only interdental bone loss was used for the screening.	• Age ≥ 18	No X-ray during the observation period recorded.	NR
Fernandes Matuck et al. [34]	Clinical study (post-mortem)	Total 7 (3 M/4 F) COVID-19 positive patients	• COVID-19 detected in 5/7 periodontal tissue samples.	• Only 7 cases. • All of these required hospitalization, even oral and nasal tubers. • Periodontal tissue could respond differently between patients with and without symptoms. • One of the non-periodontal patients was a 8- year-old child, and it could cause ACE-2 receptors to have a different expression in children.	NR	NR	Using the ACE-2 receptor.
Roganovic et al. [57]	Pre-clinical study	NR	• Diabetes and periodontitis increase microRNAs 146a and 155 in the oral cavity, upregulating ACE-2 receptors.	NR	NR	NR	MicroRNAs 146a and 155 could increase ACE-2 levels.
Sakaguchi et al. [40]	Clinical study	Tongue samples from 15 patients, average age of 53.6 years (6 M/9 F) 16 patients for gingival samples, average age of 51.88 years (6 M/10 F)	• ACE-2 and TMPRSS2 (abundant in the oral cavity) are essential molecules for SARS-CoV-2 infection. • Saliva contains many protease inhibitors that may easily reach the tongue's surface if there is removal of the tongue's coating.	NR	NR	NR	ACE-2, TMPRSS2, and furin well represented in the oral cavity.
Zhong et al. [34]	Clinical study	10 patients, average age of 53 years	• ACE-2 and furin play a crucial role for coronavirus invasion; they are mainly expressed in the epithelial cells of the oral mucosa. • ACE-2 positive and furin-positive cells were mostly localized in the epithelial layers and partly expressed in fibroblasts. • Route for SARS-CoV-2 invasion is binding to the ACE-2 receptor and fusion with the cell membrane activated by furin protease.	NR	NR	NR	ACE-2 and furin expression in the epithelial cells of the oral mucosa.

*ACE-2, angiotensin converting enzyme-2; BMI, body mass index; BoP, bleeding on probing; CAL, clinical attachment loss; CRP, C-reactive protein; COVID-19, corona virus disease 2019; GCF, gingival crevicular fluid; (M/F), male/female; NR, not reported; PI, plaque index; rRT-PCR, real-time reverse transcriptase polymerase chain reaction; SARS-CoV-2, Severe Acute Respiratory Syndrome Coronavirus-2; TMPRSS2, transmembrane protease serine 2; WBC, white blood cell*.

### Peri-Implantitis and COVID-19

Data regarding a possible link between COVID-19 and Peri-implantitis seem to be weak. Indeed, no clinical studies are present in the literature and according to the two included studies, there is not a clear and evident correlation between the diseases. According to Kadkhodazadeh et al., the global pandemic caused by COVID-19 may prevent patients from performing their regular Peri-implant and periodontal health maintenance thus the reinforcement of oral hygiene is recommended to maintain proper oral health during COVID-19 infection. [19]. In addition, we must keep in mind that the pandemic may cause psychological stress, and it may induce a worsening of periodontal or Peri-implant health [58, 59]. Furthermore, the new coronavirus has been found in the saliva of positive patients [60]. It is therefore recommended that dental procedures need to be performed in an extremely safe condition since several types of treatments may produce a large amount of aerosol and droplets mixed with the patient's saliva or even blood.

The use of hydrogen peroxide mouthwash before dental procedures and more conservative approaches (i.e., less invasive and time-consuming procedures such as manual scaling and root planing instead of ultrasonic devices) may be the suggested solutions to reduce viral spread [61, 62]. It is also important to follow infection-control protocols and personal protective measures [58].

According to Sorsa et al., the active metalloproteinase-8 point-of-care test (aMMP-8 POCT) provides a simple, Non-invasive, rapid, and real-time tool that may be used to identify the Peri-implant disease as a potential risk factor of COVID-19 [63]. The aMMP-8 levels above 20 ng/ml are indicative of active Peri-implant disease. A possible advantage of using this procedure could be the self-administration of the test and faster screening of patients. Nevertheless, future clinical studies are essential for validating this tool.

## Discussion

**Figure 2:**
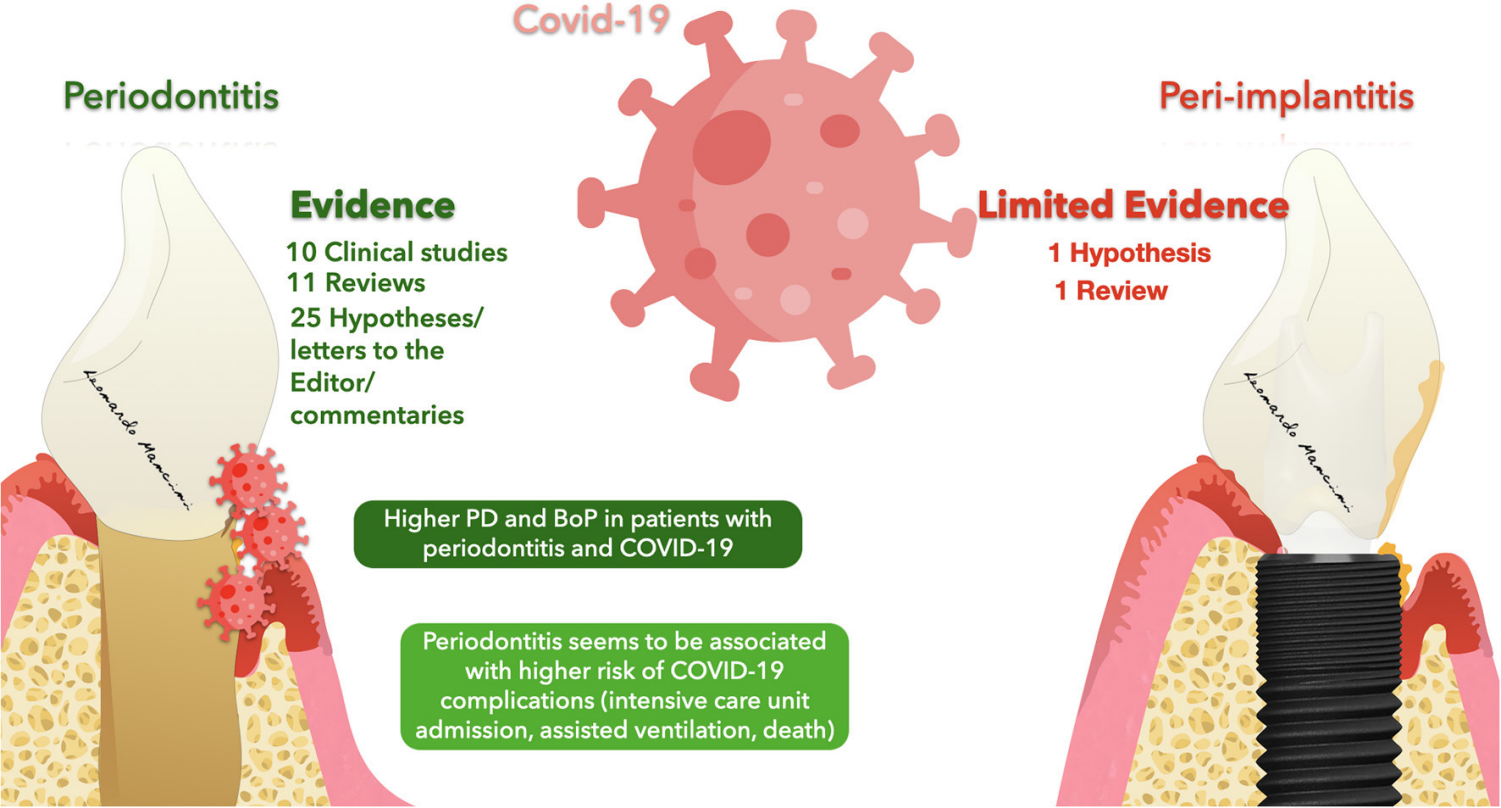
Representation of evidence regarding a possible correlation between Periodontitis/Peri-implantitis and COVID-19.

The results of this mini-review suggested the presence of several articles proposing a correlation between periodontitis or Peri-implantitis and COVID-19 (Figure 2); however, a large number of studies were predominantly hypotheses, commentaries, and simple reviews. Thus, clinical data linked to a mutual correlation between the three diseases was not truly stated. Nevertheless, 10 clinical studies were included, and through their analysis, it was possible to state a mutual correlation between COVID-19 and periodontitis. The clinical signs reported were predominantly periodontal parameters that were accentuated in the case of COVID-19 patients. As reported by Marouf et al., periodontal patients seem to have a greater probability of contracting COVID-19 [17]. The design of the clinical studies included might be a limitation due to the predominant presence of case-control and retrospective studies with a limited population. Anand et al. was the case-control study with the largest sample size of 196 patients and Larvin et al. with 13 000 patients included was the largest retrospective study [18, 56]. The molecules underlined in this review for a possible link between the diseases were predominantly ACE-2, cathepsin, Galectin-3, IL-17, NETs, and MMP-8. These molecules seem to have a crucial role in facilitating the entrance of the virus and at the same time influencing the onset and progression of the periodontal pathology. Nevertheless, scarce clinical evidence is still in the literature. Thus, a true statement regarding the molecules linked to the diseases was not feasible. Future clinical studies are needed to validate and confirm a stated correlation between the disease suggesting new therapies and protocols for treating periodontal patients affected by COVID-19 and at the same time reducing the possible exposure to the viral load and spread.

## Conclusion

The evidence regarding a mutual correlation between COVID-19 and periodontitis might be inferred from an increase in the periodontal parameters and a higher susceptibility of periodontal patients to the viral load. Nevertheless, the molecular mechanism and the possible cellular interactions need to be clarified. On the other hand, a link between COVID-19 and Peri-implantitis has not been reported due to the lack of evidence and the absence of clinical studies on the subject.

## Author Contributions

LM, LA, TP, RD, and EM: conception and design of the study, performed data analysis and interpretation, performed data acquisition, provided administrative, technical, and material support. All authors contributed to the article and approved the submitted version.

## Conflict of Interest

LM, LA, TP, and EM were employed by PerioAQ Group. The remaining author declares that the research was conducted in the absence of any commercial or financial relationships that could be construed as a potential conflict of interest.

## Publisher's Note

All claims expressed in this article are solely those of the authors and do not necessarily represent those of their affiliated organizations, or those of the publisher, the editors and the reviewers. Any product that may be evaluated in this article, or claim that may be made by its manufacturer, is not guaranteed or endorsed by the publisher.
