# Supplementary material for: Impact of COVID-19 on Periodontitis and Peri-Implantitis: A Narrative Review
Source: Front Oral Health. 2022 Feb 10;3:822824. doi: 10.3389/froh.2022.822824 (PMC8866640; doi:10.3389/froh.2022.822824)
Supplement: Supplementary file 1 [file Table_1.DOCX]

**Supplementary Table 1**. Hypothesis, commentaries, and letters to the editor regarding a possible correlation among periodontitis and COVID-19.

Abbreviations: ACE-2, Angiotensin converting enzyme-2; aMMP-8, active metalloproteinase-8; CD14+, cluster of differentiation 14; CD16+, cluster of differentiation 16; CD147, cluster of differentiation 147; Gal-3, Gelectin-3; GCF, Gingival crevicular fluid; serine 2; IL-6, interleukin 6; IL-17, interleukin 17; NETs, Neutrophil Extracellular Traps; NLRP3, NLR family pyrin domain containing 3; NPD, necrotizing periodontal disease; NR, Not reported; TMPRSS2, Transmembrane protease.

| Authors and year | Type of article | Outcomes | SARS-CoV-2 entry mechanism | possible influence on the periodontal status |
| --- | --- | --- | --- | --- |
| Badran Z. et al. 2020 [43] | Hypotheses | SARS-CoV-2 migration from gingival epithelial cells to the bloodstream or the infection of immune cells of the periodontal inflammatory infiltrate. SARS-CoV-2 in GFC could come from infected periodontal cells or through terminal capillary. | ACE-2 found in salivary glands cells, gingival and periodontal ligament fibroblasts.  Furin present in oral epithelial cells. SARS-CoV-2 can infect cells by attaching to CD147 rather than ACE-2. | in the periodontal pocket SARS-CoV-2 could find an optimal habitat for its replication.  SARS-CoV-2 might infect the oral cavity and get into the saliva or reach the bloodstream through the periodontal capillary complex.  CD147 is higher expressed in gingival epithelium of periodontal patients. |
| Balaji TM. et al. 2020 [44] | Hypotheses | Chronic periodontitis and oral cancer show higher level of protease which lead in a higher risk of Covid-19 infection | NR | NR |
| Botros et al. 2020 [13] | Short report | Improving oral health in people of any age, in particular older adults could decrease the risk of developing non oral systemic disease and could decrease the progression or occurrence of respiratory disease thus the severity and morbidity of COVID-19 | NR | Secretion of pro-inflammatory cytokines during periodontitis can promote lung adhesion and colonization by respiratory pathogens exacerbating lung infection |
| Campisi G. et al. 2021 [31] | hypotheses | possible link between chronic periodontitis and COVID-19   SARS-CoV-2 uses ACE-2 and CD147 receptors to infect and means a higher probability to be infected in periodontal patients. - overexpression of inflammatory molecules as IL-6, IL-17. | NR | NR |
| Chowdri et al. 2021 [24] | Letter to the editor | Periodontal disease causes a pro-inflammatory host response that is similar to the immune response seen in COVID-19. | NR | Diagnosis and severity of periodontal infection may directly or indirectly influence COVID-19 severity and outcome |
| Da silva ACRF et al. 2021 [32] | letter to the editor | Neutrophil Extracellular Traps (NETs) and ACE-2 play a common role in the mechanisms of action in periodontitis and COVID-19. | NR | NR |
| Elisetti N. et al. 2020 [25] | hypotheses | COVID-19 might improve cytokines levels in the periodontal pocket. | TMPRSS2 and ACE-2 detected in salivary glands | COVID-19 detected in saliva; epithelium of the periodontal pocket could be the access point. |
| Gupta S et al. 2020 [30] | Hypotheses | Increased levels of NETs in patients with COVID-19 or Periodontal disease.   Periodontitis patients might have increased risk of COVID-19 complications. | NR | Interferon alpha can stimulate the release of NETs.  NETs could play a role in the hyper-inflammatory state that occurs in advanced stages of COVID-19. |
| Hajizadeh et al 2021 [38] | Hypothesis | Similarity of cytokine expression is evident between COVID-19 and chronic periodontitis.  Moreover, the influence of gene polymorphism of cytokine on periodontal disease might play a role in COVID-19. | NR | NR |
| Jagannathan et al. 2021 [46] | Hypothesis | Patients with periodontitis have a higher risk of being infected by SARS-CoV-2; possible COVID19 complications related to the elevated levels of Furin, Cathepsin-L, Cathepsin-G in oral cavity and due to the increase of CD14+ CD16+ monocytes in peripheral blood. | ACE-2 expression in the oral mucosa with the presence of proteolytic enzymes such as Furin, Cathepsin-L and Cathepsin G | Periodontitis increases Furin, Cathepsin-L and G, CD14+ CD16+ levels and proinflammatory cytokines. |
| Kara C. et al. 2020 [45] | commentary | - link between COIVD-19 and Gal-3  - link between high level of Gal-3 and PD severity. | ACE-2 in oral mucosa  Gal-3 could enhance viral attachment and immune response | Gal-3 levels as a possible indicator of periodontitis |
| Madapusi Balaji et al. 2020 [44] | Letter to the editor | The increased protease levels and a low melatonin in chronic periodontitis and oral Cancer could increase the risk of SARS-CoV-2 infection because melatonin has anti-inflammatory, antioxidant properties and inhibits cathepsin L. | ACE-2 in the oral mucosa including tongue, and gingiva. Role of Furin, Cathepsin-L and TMPRSS2 in SARS-CoV-2 entry. | Osteopontin, Furin and Cathepsin-L levels are elevated in patients with periodontitis |
| Mancini L et al. 2020 [16] | Hypothesis | ACE-2 can have an influence in the progression of both the diseases, a mutual correlation might be suspected | ACE-2 receptors and their downregulation | Low ACE-2 levels in periodontal patients as for COVID-19 patients lead to an accentuated inflammatory pattern. |
| Monteiro Andrade R. et al. 2021 [47] | letter to the editor | Cytokine’s expression is common in periodontitis and COVID-19 | NR | NR |
| Özer Şehirli A. et al. 2021 [49] | Hypothesis | Melatonin is an anti-inflammatory and antioxidant factor that can reduce the severity of both periodontitis and covid19 preventing NLRP3 activation which increases the inflammatory process. | NR | NR |
| Park JB et al. 2020 [50] | editorial | Cannabis as an anti-inflammatory factor in both the diseases. | NR | NR |
| Patel J et a.l 2020 [51] | letter to the editor | Patients with COVID-19 have a higher risk to develop acute periodontal lesions, like necrotizing periodontal disease (NPD) due to the related bacteria co-infection particularly prevotella intermedia and many others. | NR | NR |
| Pedrosa et al 2020 [42] | Letter to the editor | Patients with diabetes and COVID-19 may present effects at the pancreas and salivary glands levels causing a qualitative and quantitative alteration in the saliva. | ACE-2 (expressed in different tissues, including salivary glands and pancreas) as receptor for entry into the target cells | NR |
| Pfützner et al. 2020 [33] | Short report | Patients with diabetes may have high risk for severe COVID-19 and at the same time they have more susceptibility to the onset of periodontitis.  Patients with diabetes need to have regular check-ups and dental hygiene treatments for monitoring and maintaining oral health. | ACE-2 highly expressed in oral cavity | Ulceration of the gingival epithelium during periodontitis may increase the risk of invasion |
| Pitones-Rubio V et al. 2020 [48] | Hypothesis | Periodontal disease may lead to severe COVID-19 due to lot of common risk factors | NR | NR |
| Raisanen IT et al. 2020 [52] | Hypothesis | aMMP-8 as screening factor for patients with severe periodontitis and positive to COVID-19. | NR | NR |
| Sahni V et al .2020 [29] | Hypothesis | Patients with severe symptoms of COVID-19 have a higher level of inflammatory cytokines. High level of IL-17 is also related in patients with gingivitis or periodontitis. | NR | NR |
| Sanz M 2021 [21] | editorial | A possible link between periodontitis and pneumonia in hospitalized patients that need assisted ventilation might cause the aspiration of l pathogens residing in the oral cavity which are increased in periodontal pockets and some of them could upregulate the ACE-2 levels in pulmonary alveolars. | NR | NR |
| Xu et al. 2020 [39] | Hypothesis | The expression of ACE-2 was higher on the epithelial cells of the tongue than on other oral sites of the oral cavity. This explains the mechanism of why the oral cavity is a potentially high-risk route for covid-19 infection. | Expression of ACE-2 on the mucosa of oral cavity (higher in tongue than buccal or gingival tissues) | NR |

**Supplementary Table 2**. Reviews and systematic reviews regarding a link between periodontitis and COVID-19.

Abbreviations: ACE-2, Angiotensin converting enzyme-2; CRP, C-reactive protein; IL1, interleukin 1; IL6, interleukin 6; IL8, interleukin 8; MMP, Matrix Metalloproteinases; NETs, Neutrophil Extracellular Traps; NR, Not reported; TMPRSS2, Transmembrane protease serine 2;

| Authors and year | Type of article | Outcomes | BIAS | SARS-CoV-2 entry mechanism | possible influence on the periodontal status |
| --- | --- | --- | --- | --- | --- |
| Aquino-Martinez R. et al. 2021 [20] | review | lung infection might be aggravated by poor oral hygiene and aspiration of periodontal bacteria. Good oral hygiene and the use of mouthrinses could prevent COVID-19 spread. | NR | NR | NR |
| Basso L. et al. 2021 [15] | Scoping review | Many papers explain how periodontitis aggravates COVID-19. The cytokine storm finded in COVID-19 patients could be supported by the cytokine release of periodontitis.  Different molecules (like osteopontin, galectin-3 and microRNA-146a and -155) are engaged in the aggravation of the disease.  Periodontal pockets as possible storage for SARS-COV-2 Periodontal pathogens increase the expression of ACE-2. | NR | NR | NR |
| Bertolini et al. 2020 [40] | Review | Crevicular fluid might be a niche for SARS-CoV-2 and could act as a potential pool for increased viral charge in the oral cavity. Periodontal treatment needs to be considered as a clinical management of covid patients, reducing furin and cathepsin-L levels. | NR | Presence of ACE-2 in the nasopharyngeal mucosa, salivary cells and oral epithelial cells.  Furin and Cathepsin-L enable SARS-CoV-2 to bind ACE-2 | Elevated levels of Furin and Cathepsin L proteases in sites with active periodontitis contribute for tissue infection and virus binding to ACE2 receptors |
| Casillas Santana MA et al 2021 [37] | Systematic review | Uncontrolled hyperglycemia (in diabetes mellitus patients) increases the risk of developing periodontitis causing ACE-2 overexpression and MMP activity in periodontal tissue. | theoretical articles and only in English | ACE-2 expressed in oral cavity (particularly in the tongue and periodontal tissue) | NR |
| Gofur 2020 [35] | Review | COVID-19 could have an impact on periodontal tissues, it causes desquamative gingivitis and oral pain. | NR | ACE-2 receptors in gingiva and oral mucosa | Periodontal pockets might be a reservoir for the virus and periodontal pathogen makes the risk of superinfection higher |
| Jafer MA. et al. 2020 [53] | Review article | Oral mucosa as an entry for coronavirus.  Periodontopathic bacteria responsible for systemic inflammation, bacteremia and pneumonia.  NETosis starts the expression of proinflammatory cytokines which leads to inflammation, and this seems to be linkable to the cytokine storms of later stages of covid. | NR | NR | NR |
| Rodean  et al 2021 [28] | Review | The inflammation is the common point that linked periodontal disease, cardiovascular disease, and COVID-19. | NR | Presence of ACE-2 in different organs | Periodontal disease increases the levels of CRP and cytokines as IL1, IL6, IL8, thus covid infection is prone to be more severe |
| Sampson et al 2020 [22] | Review | There is a link between high bacterial load in the mouth and post-viral complications.Improving oral health may reduce the risk of complications from COVID-19. | NR | NR | Cytokines of periodontitis could be aspirated and could cause inflammation or infection inside the lungs |
| Shamsoddin E E 2021 [26] | review | Shows several biases about the article Marouf N. et al 2021.  Relationships between periodontitis and severe symptoms of covid19 may exist: the inflammation of periodontitis may enhance significant covid19 symptoms in several ways. | NR | NR | NR |
| Sukumar K.et al.. 2021 [27] | Review | The main cause of the adverse events related to COVID-19 is the massive production of pro-inflammatory cytokines. Periodontal disease could increase cytokine release via different factors.  Poor oral hygiene could exaggerate SARSCoV-2 infection. | NR | NR | NR |
| Takahashi Y. et al. 2021 [23] | Review | The increased presence of periodontopathic bacteria can aggravate COVID-19 conditions with a higher ACE-2 expression.  COVID-19 aggravation may be caused by the promoted secretion of inflammatory cytokines due to aspirated periodontopathic bacteria | NR | S protein is binded to ACE-2.  S protein is cleaved by furin and TMPRSS2.  S protein could be cleaved by periodontopathic bacteria proteases. | ACE-2 expression, cytokines production in the lower lungs and the cleavage of the S protein are inducted by the aspiration of periodontopathic bacteria |
